# Adverse prognostic impact of splenectomy on survival in gastric carcinoma patients: Regression and propensity score matching analysis of 1074 patients

**DOI:** 10.1371/journal.pone.0203820

**Published:** 2018-09-11

**Authors:** Oh Jeong, Ho Goon Kim, Seong Yeob Ryu, Young Kyu Park, Mi Ran Jung

**Affiliations:** Department of Surgery, Chonnam National University School of Medicine, Jeollanam-do, South Korea; University of Texas MD Anderson Cancer Center, UNITED STATES

## Abstract

**Backgrounds:**

Patients with proximal gastric carcinoma undergo total gastrectomy with concomitant splenectomy to ensure the complete removal of splenic hilar lymph nodes. However, the impact of splenectomy on survival remains uncertain. This study aimed to investigate the impact of splenectomy on survival among patients with gastric carcinoma.

**Methods:**

Of 1074 patients who underwent total gastrectomy for proximal gastric carcinoma between 2006 and 2014, 229 patients underwent concomitant splenectomy or pancreaticosplenectomy during surgery. We investigated the prognostic impact of splenectomy using a regression and propensity score matched model.

**Results:**

The splenectomy and non-splenectomy groups differed in many baseline characteristics, including tumor stage, and had respective crude 5-year survival rates of 55% and 81% (p <0.001). In a multivariate analysis adjusted for TNM stage and other prognostic factors, splenectomy was an independent poor prognostic factor for overall survival (hazard ratio [HR] = 1.67, 95% confidence interval [CI] = 1.11–2.51) and disease-free survival (HR = 1.61, 95% CI = 1.24–2.10). A survival evaluation stratified by TNM stage showed that splenectomy adversely affected survival among patients with stage III, but not stage I, II, and IV disease. In the propensity score-matched sample, splenectomy group also showed significantly worse overall survival (5-year, 65% vs. 79%, p = 0.010) and disease-free survival (5-year, 55% vs. 72%, p = 0.025) and was an independent poor prognostic factor in a multivariate analysis adjusting TNM stage and other prognostic factors.

**Conclusions:**

Splenectomy adversely affects survival, particularly among patients with stage III gastric carcinoma, and should be avoided unless there is direct invasion to the splenic hilum.

## Introduction

Although the global incidence of gastric carcinoma is generally decreasing, the incidence of proximal gastric carcinoma, including cancer of the gastric cardia, has remained stable or increased, at least in Western countries [1]. Total gastrectomy combined with adequate lymph node (LN) dissection is currently the treatment procedure of choice for proximal gastric carcinoma, and splenectomy or pancreaticosplenectomy is performed intraoperatively when a tumor has directly invaded the spleen or pancreas. Even without such direct invasion, splenectomy is also performed to completely remove LNs at the splenic hilum as part of D2 lymphadenectomy. Although tumors rarely metastasize to the splenic hilar LNs in early-stage disease, this process occurs in up to 15–20% of patients with more advanced (i.e., stage III or higher) disease [2–4].

The impact of splenectomy on survival among patients with gastric carcinoma remains controversial. As the immunologic functions of the spleen may affect cancer growth, one might speculate that splenectomy could promote the growth of minimal residual disease during the critical early postoperative period [5]. Furthermore, some previous studies have shown that patients with metastasis to the splenic hilar LNs have a very poor prognosis even after curative surgery [2–4], and splenectomy only increases the morbidity and mortality associated with proximal gastric carcinoma without providing survival benefits [6, 7]. Still, most previous studies found that splenectomy did not affect patient survival after adjusting for other prognostic factors [8–13], and only a few studies have shown an increased risk of recurrence and death with splenectomy relative to gastrectomy alone [14–16]. Relevant previous studies of the impact of splenectomy on survival have been limited by small sample sizes and inherent selection bias. Therefore, in this study we investigated the impact of splenectomy on survival among 1074 patients who underwent total gastrectomy for proximal gastric carcinoma, using a regression and propensity score-matched analysis.

## Materials and methods

### Patients

For this retrospective case-control study, we identified 1126 patients who underwent total gastrectomy for proximal gastric carcinoma between 2006 and 2014 at our institution from a prospectively constructed gastric cancer database. Subsequently, we excluded 52 patients who had undergone preoperative chemotherapy (n = 18), had other synchronous malignancies (n = 13), or incomplete medical records (n = 21). Finally, 1074 patients were included in the analysis. This study was performed with the approval of the institutional review board at Chonnam National University, South Korea, which waived the requirement for informed consent.

All patients underwent total gastrectomy and regional LN dissection as described in the Japanese guideline for gastric cancer treatment [17]. Total gastrectomy was indicated for tumors involving the middle or upper third of the stomach, as partial gastrectomy might not achieve a sufficient resection margin. D1+ lymphadenectomy, including the perigastric (nos. 1–7) and supra-pancreatic LNs (no. 8a, 9, 11p), was performed for cT1N0 disease. D2 lymphadenectomy, which included LNs at the hepatic artery (no. 12a), splenic artery (no. 11d), and splenic hilum (no. 10) in addition to D1+ lymphadenectomy, was indicated for cT2N+ or higher disease. D2 lymphadenectomy of the no. 10 LN was performed via either the spleen-preserving method or splenectomy. At our institution, spleen-preserving D2 lymphadenectomy was the primary option; splenectomy was considered for cases involving direct tumor invasion or suspected metastasis to the no. 10 LNs. As a result, 229 (21.3%) of 1174 patients underwent splenectomy or pancreaticosplenectomy during total gastrectomy. Accordingly, patient survival outcomes were compared between the splenectomy and non-splenectomy groups in both crude and propensity score-matched samples.

### Data collection and definitions

All patient data pertaining to demographics, operative results, and clinicopathological characteristics were retrieved from the prospectively collected database. Demographic data included age, sex, body mass index, comorbidities, and American Society of Anesthesiologists (ASA) classification. Operative data included curability, operative approach, extent of lymph node dissection, combined resection, operating time, and operative bleeding. Pathological data included tumor location, tumor size, differentiation, Lauren classification, tumor invasion, nodal metastasis, and distant metastasis. Information about postoperative outcomes, including morbidity, mortality, and hospital stay, were also collected. Postoperative morbidity and mortality were defined as the occurrence of complications or death, respectively, within 30 days after surgery. Pathological staging was based on the seventh edition of the International Union for Cancer Control (UICC)/American Joint Committee on Cancer (AJCC) tumor node metastasis (TNM) classification of gastric carcinoma [18].

Patient survival was defined as the time interval from surgery to death from gastric cancer. Deaths from other causes were regarded as censored data. Patient survival was ascertained by December 2016, and censored patients were followed for a median of 52 months (range: 19–108 months).

### Propensity score matching

To adjust for systemic differences in baseline characteristics between the two groups, patients were matched using the propensity score-matching method [19]. To generate propensity scores, we selected matching variables that are regarded as having a correlation with survival outcomes or treatment decision. Consequently, propensity scores were generated using a binary logistic regression model that incorporated the extent of lymph node dissection, tumor location, tumor size, histological differentiation, and TNM stage. After generating the propensity score, patients in the splenectomy group were matched at a 1:1 ratio using the nearest neighborhood matching method with a caliper of 0.05. After matching, the quality of the matching results was evaluated using the propensity score distribution and standardized differences in the matching variables before and after matching.

### Statistical methods

Continuous variables were compared using the t-test, and categorical variables were compared using the chi-square test. In the crude sample, survival curves were plotted according to the Kaplan–Meier method and compared using the log-rank test. The Cox proportional hazard model was used to generate a multivariate prognostic model for which the proportionality assumption was ascertained using a log-log survival plot. In the multivariate analysis, multicollinearity of independent variables were checked calculating the tolerance and variance inflation factor (VIF).

In the propensity score-matched sample, survival was compared between of matched groups using the stratified log-rank test (Prentice–Wilcoxon test), which accounts for the nature of matched data [20]. All statistical analyses were performed using SPSS, version 23.0 (IBM Corp., Armonk NY., USA) and R 3.1.0 (R Project for Statistical Computing, Vienna, Austria). For all analyses, a two-sided P value of <0.05 was considered statistically significant.

## Results

### Patient characteristics

The crude sample included 229 patients in the splenectomy group and 845 patients in the non-splenectomy group. The crude sample showed systemic differences in baseline characteristics, including the operative methods and pathological outcomes between the two groups (Table 1). Patients in the splenectomy group had undergone open surgery (97.8% vs. 71.4%) and D2 lymph node dissection (93.0% vs. 51.7%) more frequently than the non-splenectomy group. Furthermore, the splenectomy group was significantly more likely to have an advanced pTNM stage, compared to those in the non-splenectomy group (p <0.001). Regarding operative outcomes, the splenectomy group had a significantly longer operating time (242 vs. 228 min, p = 0.010), larger operative bleeding (325 vs. 203 ml, p < 0.001), increased morbidity (29.3% vs. 19.8%, p <0.001), and longer hospital stay (14.4 vs. 10.4 days, p <0.001) than the non-splenectomy group.

**Table 1:** Baseline characteristics of patients before and after matching.

	Total population			Propensity-matched population		
	Splenectomy (n = 229)	No-splenectomy (n = 845)	P	Splenectomy (n = 97)	No-splenectomy (n = 97)	P
Age (years)	62.1 ± 11.6	60.8 ± 12.3	0.130	61.5 ± 12.0	62.3 ± 11.5	0.492
Sex			0.654			0.754
Male	155 (67.7)	585 (69.2)		69 (71.1)	67 (69.1)	
Female	74 (32.3)	260 (30.8)		28 (28.9)	30 (30.9)	
BMI (kg/m^2^)	22.3 ± 3.0	23.2 ± 3.2	< 0.001	22.6 ± 3.0	22.7 ± 3.2	0.927
ASA status			0.016			0.781
1	55 (24.2)	287 (34.2)		19 (19.6)	21 (21.7)	
2	158 (69.6)	510 (60.8)		77 (79.4)	74 (76.3)	
3	14 (6.2)	42 (5.0)		1 (1.0)	2 (2.1)	
Operative approach			< 0.001			0.282
Open	224 (97.8)	603 (71.4)		92 (34.8)	87(89.7)	
Laparoscopy	5 (2.2)	242 (28.6)		5 (5.2)	10 (10.3)	
Lymph node dissection			< 0.001			1.000
< D2	16 (7.0)	408 (48.3)		6 (6.2)	6 (6.2)	
D2	213 (93.0)	437 (51.7)		91 (93.8)	91 (93.8)	
Tumor location			< 0.001			1.000
Middle	89 (38.9)	234 (27.7)		32 (33.0)	32 (33.0)	
Upper	108 (47.2)	559 (66.2)		59 (50.8)	60 (61.9)	
Whole stomach	32 (14.0)	52 (6.2)		6 (6.2)	5 (5.2)	
Tumor size (cm)	7.7 ± 3.9	4.9 ± 3.6	< 0.001	6.1 ± 3.0	6.3 ± 2.9	0.674
Differentiation			0.005			0.369
Diff	63 (27.5)	316 (37.4)		33 (34.0)	26 (26.8)	
Undiff	166 (72.5)	529 (62.6)		64 (66.0)	71 (73.2)	
Tumor invasion*			< 0.001			0.933
pT1	18 (7.9)	359 (42.5)		14 (14.4)	14 (14.4)	
pT2	7 (3.1)	113 (13.4)		3 (3.1)	5 (5.2)	
pT3	58 (25.3)	150 (17.8)		32 (33.0)	32 (33.0)	
pT4	146 (63.8)	223 (26.4)		48 (49.5)	46 (47.4)	
Nodal metastasis*			< 0.001			0.895
pN0	44 (19.2)	517 (61.2)		25 (25.8)	26 (26.8)	
pN1	38 (16.6)	96 (11.4)		20 (20.6)	17 (17.5)	
pN2	42 (18.3)	84 (9.9)		22 (22.7)	20 (20.6)	
pN3	105 (45.9)	148 (17.5)		30 (30.9)	34 (35.1)	
TNM stage*			< 0.001			1.000
I	23 (10.0)	420 (49.7)		16 (16.5)	16 (16.5)	
II	33 (14.4)	173 (20.5)		18 (18.6)	18 (18.6)	
III	108 (47.2)	163 (19.3)		51 (52.6)	50 (51.5)	
IV	65 (28.4)	89 (10.5)		12 (12.4)	13 (13.4)	

Data are expressed as means ± standard deviations or n (%).

*Pathological stages are based on the seventh edition of the AJCC/UICC classification of malignant tumors.

ASA status, American Society of Anesthesiologists physical status classification system; BMI, body mass index

**Fig 1:**
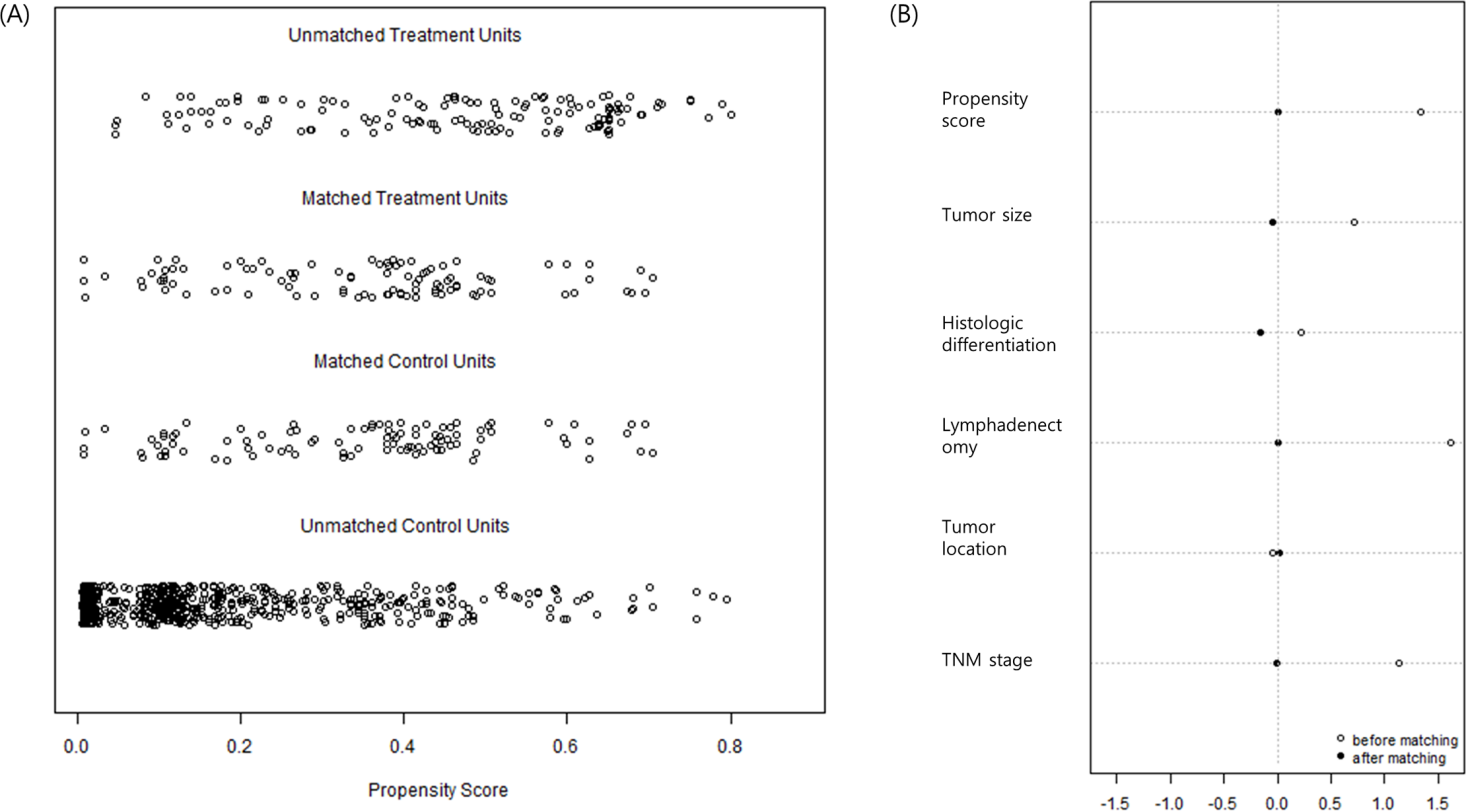
Distribution of propensity scores (A) and standardized differences in matching variables (B). After matching, two groups showed very similar propensity score distribution and minimized standardized differences in variables.

Propensity score matching generated 97 patients from each group. Fig 1 shows very similar distributions of propensity scores between the two groups (Fig 1A) and minimized standardized differences in the matched variables (Fig 1B) after matching. The baseline characteristics, including demographic and pathological outcomes, of the matched samples became well balanced after matching (Table 1).

### Survival analysis in the crude sample

The 5-year survival rates for the splenectomy and non-splenectomy groups were 55% and 81% for overall survival (log-rank p <0.001) and 49% and 80% for disease free survival (log-rank p <0.001), respectively. To investigate the impact of splenectomy on patient survival, 920 patients who underwent curative surgery were included in the survival analysis. In a univariate analysis, age, tumor location, lymphovascular invasion, Lauren classification, tumor size, tumor invasion (pT), nodal metastasis (pN), and splenectomy were found to significantly associate with overall survival. In a multivariate analysis of these factors, splenectomy (hazard ratio [HR] = 1.67, 95% confidence interval [CI] = 1.11–2.51), age, lymphovascular invasion, tumor invasion, and nodal metastasis remained independent poor prognostic factors (Table 2). Splenectomy was also an independent poor prognostic factor (HR = 1.61, 95% CI = 1.24–2.10) for disease free survival, along with Lauren type, tumor invasion, and nodal metastasis. All the variables in the multivariate model showed appropriate range of tolerances (0.47 to 0.93) and VIFs (1.1 to 2.2), which indicate no significant risk of multicollinearity.

**Table 2:** Univariate and multivariate analyses of prognosis in the crude sample.

	Univariate		Multivariate	
	HR (95% CI)	P	Adjusted HR (95% CI)	P
Age (years)	1.02 (1.00–1.04)	0.021	1.03 (1.01–1.04)	0.002
Sex (male)	1.50 (0.98–2.29)	0.060		
Tumor location (whole stomach)	3.94 (2.25–6.89)	< 0.001	1.40 (0.73–2.69)	0.314
Differentiation (undifferentiated)	1.41 (0.96–2.07)	0.081		
Lymphovascular invasion	3.46 (2.39–5.02)	< 0.001	1.57 (1.01–2.45)	0.046
Lauren classification (diffuse)	1.76 (1.19–2.58)	0.004	1.50 (0.98–2.32)	0.064
Tumor size (cm)	1.16 (1.01–1.21)	< 0.001	0.99 (0.94–1.06)	0.932
No. harvested LN	0.99 (0.98–1.01)	0.561		
Tumor invasion (vs. pT1)				
pT2	1.57 (0.71–3.49)	0.268	1.27 (0.55–2.89)	0.574
pT3	3.46 (1.94–6.16)	< 0.001	1.61 (0.80–3.24)	0.181
pT4a	6.86 (4.01–11.66)	< 0.001	2.24 (1.08–4.64)	0.030
pT4b	4.84 (1.43–16.45)	0.011	1.27 (0.33–4.90)	0.727
pN (vs. pN0)				
pN1	2.79 (1.59–4.90)	< 0.001	1.60 (0.85–3.00)	0.143
pN2	3.53 (2.05–6.08)	< 0.001	1.66 (0.87–3.17)	0.124
pN3a	5.66 (3.35–9.54)	< 0.001	2.64 (1.38–5.07)	0.003
pN3b	12.40 (7.17–21.43)	< 0.001	4.01 (1.91–8.40)	< 0.001
Postoperative morbidity	1.28 (0.83–1.97)	0.259		
Splenectomy	3.42 (2.37–4.94)	< 0.001	1.67 (1.11–2.51)	0.015

HR, hazard ratio; CI, confidence interval, LN, lymph nodes

### Impact of splenectomy on different tumor stages

**Fig 2:**
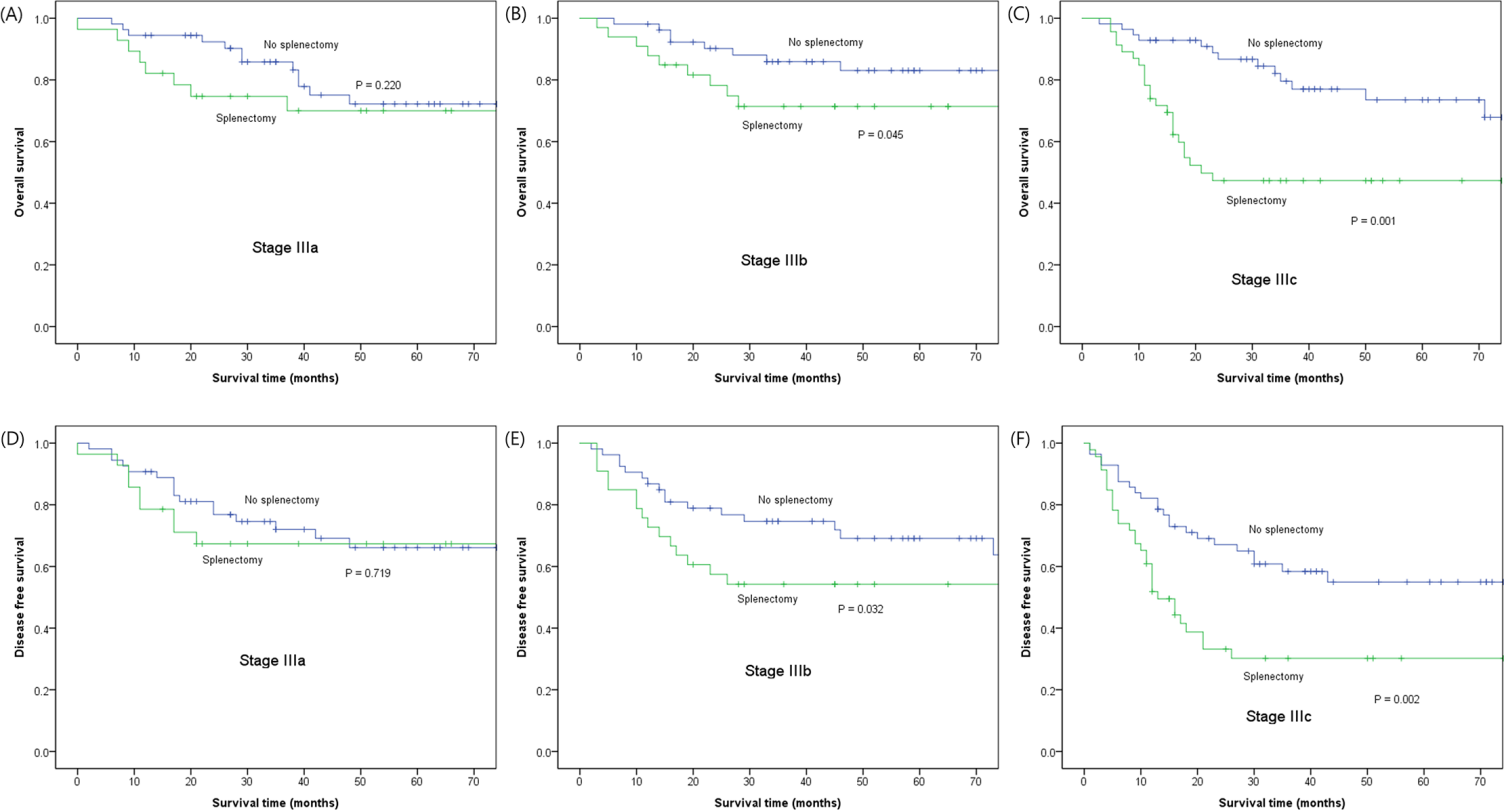
Comparison of overall (A, B, and C) and disease-free survival (D, E, and F) between splenectomy and non-splenectomy groups in stage III patients of crude sample.

To investigate the impact of splenectomy on different tumor stages, splenectomy and non-splenectomy patients were compared within subgroups stratified by pTNM stage (Fig 2). Although splenectomy did not influence patient survival in the stage I, II, and IV groups, patients with stage III disease who had undergone splenectomy showed a significantly worse overall survival (Fig 2A, 2B and 2C) and disease free survival (Fig 2D, 2E and 2F), compared to their non-splenectomy counterparts.

Table 3 presents the results of univariate and multivariate analyses of prognostic factors affecting stage III patients. In addition to splenectomy, the tumor location, tumor size, lymphovascular invasion, and nodal metastasis were all significantly associated with overall survival in the univariate analysis. A multivariate analysis revealed that splenectomy (HR = 2.18, 95% CI = 1.36–3.50), lymphovascular invasion (p = 0.057), and nodal metastasis (p = 0.029) remained independent poor prognostic factors for overall survival in patients with stage III disease. As for disease free survival, splenectomy (HR = 1.79, 95% CI = 1.21–2.63) and nodal metastasis (HR = 1.73, 95% CI = 1.14–2.62) were independent poor prognostic factor in stage III patients. Multicollinearity test for the variables in the multivariate model showed appropriate range of tolerances and VIFs.

**Table 3:** Univariate and multivariate analyses of prognosis among stage III patients.

	Univariate		Multivariate	
	HR (95% CI)	P	Adjusted HR (95% CI)	P
Age (years)	1.00 (0.99–1.02)	0.334		
Sex (male)	1.33 (0.81–2.19)	0.266		
Tumor location (whole stomach)	2.33 (1.28–4.25)	0.006	1.59 (0.81–3.09)	0.171
Differentiation (undifferentiated)	1.13 (0.61–2.10)	0.691		
Lauren classification (diffuse)	1.54 (0.96–2.45)	0.070		
Tumor size (cm)	1.08 (1.01–1.14)	0.021	1.02 (0.95–1.10)	0.526
Lymphovascular invasion	1.98 (1.10–3.54)	0.022	1.77 (0.98–3.20)	0.057
Tumor invasion (pT1-3 vs. pT4)*	1.39 (0.84–2.33)	0.202		
Nodal metastasis (pN0-2 vs. pN3)*	2.00 (1.25–3.21)	0.004	1.74 (1.05–2.87)	0.029
Splenectomy	2.29 (1.45–3.63)	< 0.001	2.18 (1.36–3.50)	0.001

HR, hazard ratio; CI, confidence interval

*pT1-3 and pN0-2 were analyzed as one group because of small number of patients with pT1-2 or pN0-1 in stage III.

### Impact of splenectomy on survival among patients with no. 10 LN metastasis

Splenectomy is generally performed with the intent to improve survival by completely resecting no. 10 LN metastases. Therefore, we compared the survival outcomes of 63 patients (5.9%) with no. 10 LN metastasis who had or had not undergone splenectomy. The 45 patients in this group who underwent splenectomy had significantly worse survival outcomes (5-year survival, 22% vs. 42% for non-splenectomy, log-rank p = 0.024). The univariate and multivariate analyses of prognostic factors identified tumor size and TNM stage, but not splenectomy, as independent prognostic factors among these patients (Table 4).

**Table 4:** Univariate and multivariate analyses of prognosis among patients with no.10 LN metastasis.

	Univariate		Multivariate	
	HR (95% CI)	P	Adjusted HR (95% CI)	P
Age (years)	0.99 (0.97–1.02)	0.515		
Sex (male)	0.64 (0.33–1.24)	0.188		
Tumor location (whole stomach)	1.39 (0.65–2.98)	0.395		
Differentiation (undifferentiated)	1.29 (0.45–3.69)	0.527		
Lauren classification (diffuse)	1.17 (0.61–2.27)	0.634		
Tumor size (cm)	1.13 (0.14–1.22)	0.002	1.12 (1.03–1.22)	0.005
Lymphovascular invasion	1.15 (0.41–3.26)	0.788		
TNM stage (III vs. IV)	2.82 (1.41–5.68)	0.004	2.86 (1.40–5.84)	0.004
Splenectomy	2.79 (1.08–7.19)	0.034	1.69 (0.62–4.60)	0.299

HR, hazard ratio; CI, confidence interval

### Survival analysis of the propensity score-matched sample

**Fig 3:**
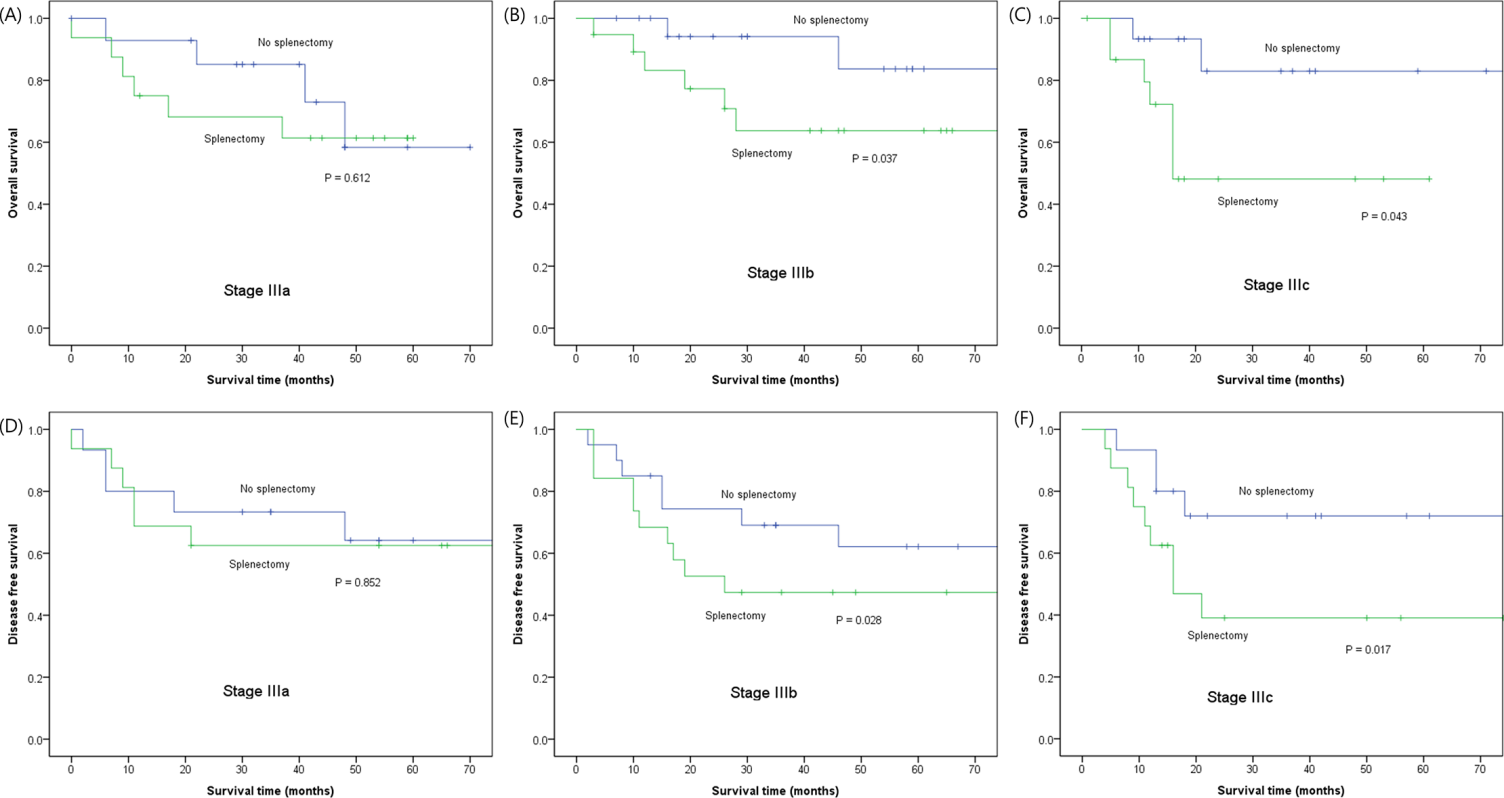
Comparison of overall (A, B, and C) and disease-free survival (D, E, and F) between splenectomy and non-splenectomy groups in stage III patients of propensity score matched sample.

To further investigate the survival impact of splenectomy, we performed survival analysis in propensity score matched sample. In a survival comparison within the matched sample, splenectomy was associated with significantly worse overall (5-year survival, 65% vs. 79%, Prentice–Wilcoxon p = 0.010) and disease free survival (5-year survival, 55% vs. 72%, Prentice-wilcoxon p = 0.025), compared to non-splenectomy. In a subgroup analysis by pTNM stage, splenectomy again was associated with significantly worse survival among patients with stage III disease (Fig 3), but not in stage I, II, and IV. Univariate and multivariate analysis of prognostic factors showed that splenectomy was an independent poor prognostic factor (HR = 2.45, 95% CI = 1.31–4.62) for overall survival when adjusting TNM stage and other prognostic factors (Table 5). As for disease-free survival, splenectomy (HR = 1.89, 95% CI = 1.15–3.10) and TNM stage were independent poor prognostic factors for disease-free survival.

**Table 5:** Univariate and multivariate analysis of prognostic factors for overall survival in matched sample.

	Univariate		Multivariate	
	HR (95% CI)	P	Adjusted HR (95% CI)	P
Tumor location (whole stomach)	3.64 (1.53–8.63)	0.003	0.74 (0.23–2.38)	0.617
Differentiation (undifferentiated)	2.69 (1.25–5.76)	0.001	1.41 (0.61–3.30)	0.420
Tumor size (cm)	1.23 (1.22–1.35)	< 0.001	1.09 (0.95–1.26)	0.184
Lymphovascular invasion	2.01 (1.07–3.76)	0.029	1.69 (0.87–3.29)	0.120
TNM stage (vs. stage I)				
Stage II	0.86 (0.12–6.12)	0.882	0.49 (0.65–3.83)	0.504
Stage III	5.57 (1.32–23.48)	0.019	2.72 (0.56–13.22)	0.214
Stage IV	28.92 (6.46–129.37)	< 0.001	13.07 (2.45–69.54)	0.003
Splenectomy	2.18 (1.18–4.02)	< 0.013	2.45 (1.31–4.62)	0.005

HR, hazard ratio; CI, confidence interval

## Discussion

Splenectomy is frequently performed during total gastrectomy with the intent to completely remove the splenic hilar lymph nodes. However, the impact of splenectomy on survival among patients with gastric carcinoma remains controversial. As noted previously, although many studies reported increased morbidity and mortality, the effects of splenectomy on both tumor recurrence and survival have not been clearly defined [8–13]. The present study is the largest to demonstrate the adverse prognostic impact of splenectomy in a cohort of patients with gastric carcinoma. The large sample size allowed us to obtain robust results by subjecting both crude and propensity score-matched samples to a systemic survival analysis. Accordingly, we demonstrated that splenectomy not only increased postoperative morbidity but also adversely affected long-term survival, particularly among patients with stage III disease, in both samples. Therefore, our results suggest that splenectomy should be avoided except in cases involving direct tumor invasion to the splenic hilum.

In contrast, many previous studies have failed to demonstrate a survival impact of splenectomy after adjusting for other prognostic factors [8–13]. However, most of these previous studies were limited by an insufficient sample size and thus could not clearly demonstrate the survival impact of splenectomy. In fact, many studies reported a tendency of a lower survival rate among patients who underwent splenectomy for advanced-stage (stage III or higher) disease, but failed to demonstrate statistical significance. Ito et al. [13] reported that patients who underwent splenectomy for pT3–4 disease tended to have poor overall survival outcomes, although the comparison did not reach statistical significance. Furthermore, a recent large randomized trial in Japan demonstrated that gastrectomy alone was not inferior to splenectomy in terms of survival. However, a subgroup analysis from that trial observed a tendency toward lower survival rates with splenectomy compared to gastrectomy alone among patients with ≥pT3 and ≥pN2 disease [6]. Similar findings were also observed in other studies [9–11].

Meanwhile, Fatours et al. [14] identified splenectomy as an independent poor prognostic factor for recurrence and survival among patients undergoing curative surgery, after adjusting for nodal metastasis and tumor invasion. In a large retrospective study of 3477 patients undergoing curative gastrectomy, an analysis of survival stratified by TNM stage indicated significantly reduced survival among patients with stage II and III disease, but not among those with stage I or IV disease [15]. Griffith et al. [16] also demonstrated significantly worse survival among patients who underwent splenectomy for stage III disease, and identified this procedure as an independent prognostic factor in a multivariate survival analysis. Consistent with these earlier findings, our large-cohort study confirmed the poor prognostic impact of splenectomy, especially for advanced-stage gastric cancer.

In this study, we used propensity score matching as well as a regression analysis to evaluate patient survival. As with other studies, the tendency to perform splenectomy for more advanced disease led to an inherent selection bias that resulted in a systematic difference in the distribution of baseline characteristics between patients who had and had not undergone splenectomy. In this setting, propensity score matching can be effectively used to remove the effects of confounding by comparing outcomes between matched subjects with similarly distributed baseline characteristics [19]. The use of both the regression and matched analyses ensures robust results, especially when the results of two methods coincide. In the present study, our matched sample demonstrated a good matching quality, as reflected by the similar propensity score differences and minimization of standardized differences after matching (Fig 2). Accordingly, our propensity score-matched sample reaffirmed the finding that splenectomy was a poor prognostic factor in the multivariate regression analysis of our crude sample.

The spleen is the largest lymphoid organ in our body; in addition to its capacity as a reservoir, it performs major functions related to hemofiltration, immunity, and hematopoiesis. Accordingly, splenectomy is associated with both infectious and thrombotic complications [21]. In addition, the potential for asplenia to promote increased cancer growth and recurrence has been the subject of considerable investigation. In some animal studies, splenectomy was associated with a significant increase in malignant tumor induction, along with a decrease in the peripheral blood lymphocyte count after tumor inoculation [22, 23]. In some epidemiologic studies [24, 25] (but not all [26]), splenectomy was associated with an excess risk of carcinogenesis. In a recent large study of 8149 U.S. veterans who underwent splenectomy, the risk of certain solid tumors, including esophageal, liver, colon, pancreas, lung, prostate, and hematologic malignancies, significantly increased during a follow-up of up to 27 years [27]. These functions of the spleen might explain the decreased survival observed among patients who underwent splenectomy for gastric cancer. Like the role of splenectomy in human carcinogenesis, however, the mechanisms by which the spleen (or lack thereof) influences tumor development or growth remain to be elucidated.

In the present study, splenectomy influenced survival only among patients with stage III disease, consistent with similar findings in other studies [15, 16]. This might be attributed to the generally excellent long-term survival and rarity of disease recurrence among patients with stage I or II disease. On the other hand, patients with stage IV disease tend to have very poor survival outcomes, regardless of splenectomy. However, as disease recurrence is relatively common among stage III patients, splenectomy may accelerate the growth of residual tumors and decrease patient survival. This possibility may warrant further investigation in a randomized clinical trial of stage III patients.

Splenectomy is generally performed with the intent to improve survival by completely resecting metastatic no. 10 LNs. However, previous studies have shown that spleen-preserving techniques no. 10 LN resection are oncologically as feasible as splenectomy [28]. Our study also demonstrated that splenectomy did not improve survival when compared with a spleen-preserving technique, even in cases involving no. 10 LN metastasis. Therefore, we believe that the evidence does not support prophylactic splenectomy in the context of D2 lymphadenectomy. Furthermore, a recent large randomized trial in Japan reported that total gastrectomy alone (i.e., without no. 10 LN resection/less than D2) is not inferior to total gastrectomy plus splenectomy in terms of survival [6]. This suggests that no. 10 LN dissection may not be essential for the treatment of proximal gastric carcinoma. This evidence is expected to change the current surgical strategy for proximal gastric carcinoma in our region.

This study had some limitations of note. First, it did not address information about disease recurrence. The association of different patterns of recurrence with splenectomy might provide further insights regarding the influence of splenectomy on tumor growth. Second, although propensity score matching might resolve selection bias, this method introduces other potential sources of bias such as the risk of unmeasured confounding, inappropriate matching quality, or loss of data. The propensity score should therefore be considered a supplement to traditional methods when estimating the effects of treatments in observational studies. The results of this study should be confirmed in a randomized large-scale clinical trial.

## Conclusions

The present study has identified splenectomy as a poor prognostic factor in a large-cohort study via regression and propensity score matched analyses. Notably, splenectomy significantly affected patient survival among stage III patients. We therefore recommend that splenectomy should be avoided except in cases of direct tumor invasion or metastasis to the splenic hilum. Finally, our findings warrant a large-scale randomized trial focused on stage III patients to investigate the survival impact of splenectomy.
